# Coronary Microvascular Dysfunction in Takotsubo Syndrome: Subtype-Specific Patterns Revealed by Invasive Assessment

**DOI:** 10.3390/diagnostics16152380

**Published:** 2026-07-29

**Authors:** Vojtech Brazdil, Jiri Parenica, Martin Hudec, Martin Poloczek, Jan Kanovsky, Roman Stipal, Petr Jerabek, Otakar Bocek, Jiri Jarkovsky, Andrea Kyselova, Petr Kala

**Affiliations:** 1 Department of Internal Medicine and Cardiology, University Hospital Brno, Jihlavska 20, 625 00 Brno, Czech Republic; parenica.jiri@fnbrno.cz (J.P.); hudec.martin@fnbrno.cz (M.H.); poloczek.martin@fnbrno.cz (M.P.); kanovsky.jan@fnbrno.cz (J.K.); stipal.roman@fnbrno.cz (R.S.); jerabek.petr@fnbrno.cz (P.J.); bocek.otakar@fnbrno.cz (O.B.); kalapetr7@gmail.com (P.K.); 2 Faculty of Medicine, Masaryk University, Kamenice 5, 625 00 Brno, Czech Republic; 3 Institute of Health Information and Statistics of the Czech Republic, Palackého náměstí 4, 128 01 Prague, Czech Republic; jiri.jarkovsky@uzis.cz (J.J.); andrea.kyselova@uzis.cz (A.K.); 4 Institute of Biostatistics and Analyses, Faculty of Medicine, Masaryk University, Kamenice 5, 625 00 Brno, Czech Republic

**Keywords:** Takotsubo syndrome, coronary microvascular dysfunction, invasive coronary physiology, index of microcirculatory resistance, coronary flow reserve

## Abstract

**Background/Objectives:** Coronary microvascular dysfunction (CMD) is considered a key mechanism of Takotsubo syndrome (TTS), but its phenotype-specific territorial distribution remains unclear. This study investigated CMD within the left anterior descending artery (LAD) and left circumflex/obtuse marginal/ramus intermedius (LCx/OM/RI) territories in apical (AP) and midventricular (MV) TTS. **Methods:** In this prospective single-centre study, 31 patients with acute TTS were enrolled (median age, 71 years; 93.5% women; 20 AP and 11 MV). During the acute phase, coronary flow reserve (CFR), index of microcirculatory resistance (IMR), and fractional flow reserve (FFR) were assessed invasively in both territories. CMD was defined as CFR ≤ 2 and/or IMR > 25; the neutral descriptive subtypes Pattern A (CFR ≤ 2 with IMR ≤ 25) and Pattern B (CFR ≤ 2 with IMR > 25) were distinguished. **Results:** FFR was ≥0.80 in all patients, excluding haemodynamically significant epicardial disease; at least one abnormal microvascular parameter was present in 26/31 patients (83.9%). LAD-CFR was significantly lower in AP than in MV TTS (1.6 vs. 2.6; *p* = 0.003), whereas LCx/OM/RI-CFR did not differ (1.6 vs. 1.5; *p* = 0.679). Bifocal CFR reduction predominated in AP TTS (55.0% vs. 9.1%; *p* = 0.020), whereas isolated LCx/OM/RI involvement occurred only in MV TTS (45.5% vs. 0.0%; *p* = 0.003). Pattern B in the LAD territory was more common in AP TTS (50.0% vs. 9.1%; *p* = 0.047). **Conclusions:** In this cohort, TTS showed a phenotype-specific territorial distribution of CMD: the apical phenotype predominantly involved Pattern B in the LAD territory, whereas the midventricular phenotype showed isolated LCx/OM/RI involvement within the interrogated left coronary circulation. These hypothesis-generating findings suggest phenotype-associated differences that require confirmation in larger, adequately powered cohorts.

## 1. Introduction

Takotsubo syndrome (TTS) is an acute, reversible dysfunction of the left ventricle (LV) that mimics acute coronary syndrome (ACS) in its clinical presentation. Due to overlapping symptoms, such as sudden chest pain, dyspnoea or palpitations, differentiating TTS from ACS can be challenging. In more severe cases, TTS may manifest as ventricular arrhythmias or cardiogenic shock. As in ACS, elevated cardiac biomarkers and electrocardiographic (ECG) changes are often present in TTS, further complicating the diagnosis [1,2].

Approximately 1–2% of patients evaluated for suspected ACS receive a final diagnosis of TTS [3]. Recent studies indicate that both the acute and long-term prognosis of patients with TTS may be worse than previously assumed [4,5,6]. According to data from large registries, in-hospital mortality during the acute phase is approximately 4% [7]. The primary diagnostic tools for TTS remain acute echocardiography and coronary angiography, which demonstrate normal epicardial coronary arteries in most patients. However, approximately 15% of patients may have concomitant ischaemic heart disease, with the LV wall-motion abnormality typically extending beyond the territory corresponding to the lesion [4].

TTS is often triggered by an emotional stressor, either negative or positive, or by physical stress (exacerbation of a chronic condition, neurological disorders, infections, surgery, and others). TTS typically affects postmenopausal women, with the average age ranging between 67 and 70 years; women account for up to 90% of all cases [3,4]. Data from the International Takotsubo Registry show that an identifiable trigger is present in the majority of patients, most commonly a physical stressor. However, in roughly one-third of patients, the trigger remains unclear [4].

The pathophysiology of TTS remains incompletely understood and is likely multifactorial. Excessive catecholamine-mediated activation of the sympathetic nervous system is considered a common denominator of the underlying pathophysiological processes, triggering a cascade of adverse effects on cardiomyocytes and the coronary circulation. The proposed mechanisms include coronary microvascular dysfunction (CMD), multivessel epicardial coronary spasm, transient ischaemia caused by atherosclerotic plaque rupture, and direct catecholamine-mediated myocardial toxicity [1,8,9].

CMD comprises a heterogeneous spectrum of structural and functional abnormalities of the microcirculation that lead to impaired coronary blood flow and, consequently, to an imbalance between myocardial oxygen supply and demand [10]. A growing body of evidence suggests that CMD may play a key role in the pathophysiology of TTS, particularly during the acute phase of the disease. In the context of invasive assessment, two CMD subtypes—functional and structural microvascular dysfunction—are recognised within the INOCA framework according to the combination of coronary flow reserve (CFR) and the index of microcirculatory resistance (IMR); because this classification has not been validated in TTS, we adopt the neutral descriptive terms Pattern A and Pattern B (defined in Section 2.2) [11,12]. The nature of these microcirculatory patterns in TTS nevertheless remains poorly understood and underinvestigated.

The aim of this study was to provide a detailed invasive assessment of microvascular function in patients with TTS, specifically within individual territories of the left coronary artery. Particular attention was paid to comparing findings across different TTS phenotypes to determine whether distinct forms of the syndrome exhibit specific microvascular abnormalities. A secondary objective was to identify the two CMD patterns—Pattern A and Pattern B—and to assess their prevalence across individual TTS phenotypes. Given the limited sample size, this study was conceived as a hypothesis-generating, exploratory investigation.

## 2. Materials and Methods

### 2.1. Study Design and Population

This prospective single-centre study aimed to evaluate coronary microcirculation in patients with TTS and was conducted at University Hospital Brno, Brno, Czech Republic. The study complied with the Declaration of Helsinki and was approved by the local ethics committee (University Hospital Brno, approval number 13-070218, approval date 7 March 2018). All patients provided written informed consent.

A total of 31 clinically stable patients with suspected ACS or TTS were enrolled. These patients showed no evidence of obstructive coronary artery disease on cardiac catheterisation and fulfilled the InterTAK Diagnostic Criteria for TTS [1]. In all enrolled patients, invasive assessment of coronary microcirculation in the LAD and LCx/OM/RI territories was performed immediately after the initial coronary angiography, including measurements of CFR, IMR, and FFR (see the following section for detailed methodology). Left ventricular angiography was performed as part of the catheterisation procedure in all patients to assess the TTS phenotype—apical (AP) or midventricular (MV). In addition, initial echocardiography was performed in all enrolled patients 1–3 days after catheterisation. Scheduled clinical follow-up, including repeat echocardiography, was planned for all 31 enrolled patients; patients who did not attend and for whom no follow-up information was available were classified as lost to follow-up. Clinical events, including death, rehospitalisation for cardiovascular reasons, acute myocardial infarction (AMI), and repeat coronary angiography (CAG), were monitored [13].

### 2.2. Assessment of Coronary Microcirculation

All patients underwent invasive functional assessment of the coronary circulation in the LAD and LCx/OM/RI territories. This assessment was performed after initial catheterisation, during which obstructive coronary artery disease was ruled out. All measurements were performed using a PressureWire pressure–temperature sensor-tipped guidewire (Abbott; formerly St. Jude Medical, St. Paul, MN, USA) and a RadiAnalyzer Xpress analyser (Abbott, St. Paul, MN, USA), employing the bolus thermodilution technique. To determine resting coronary flow, 3 mL of room-temperature saline was rapidly administered three times. Measurements were performed during maximal hyperaemia induced by continuous intravenous infusion of adenosine (Adenocor; Sanofi, Prague, Czech Republic), at the standard recommended dose of 140 μg/kg/min. These measurements were performed sequentially in the LAD and in the selected LCx/OM/RI vessel, with the guidewire positioned in the distal segment of each interrogated vessel. Based on these measurements, the relevant indices for assessing coronary physiology were derived. FFR, calculated as the ratio of distal coronary pressure to aortic pressure during maximal hyperaemia, was used to assess functional impairment of the epicardial circulation [14]. CFR, an index reflecting both epicardial flow and coronary microcirculation, was calculated as the ratio of mean transit time at rest to mean transit time during maximal hyperaemia. Because epicardial flow was preserved in all patients (FFR ≥ 0.80), CFR values in this cohort primarily reflected coronary microcirculatory function. IMR was calculated as the product of distal coronary pressure and mean hyperaemic transit time and was used to quantitatively assess coronary microcirculatory function independently of the epicardial circulation [15]. CFR ≤ 2 and/or IMR > 25 were considered pathological and indicative of impaired coronary microcirculation. These thresholds are consistent with consensus recommendations for the invasive assessment of CMD in patients with ischaemia with non-obstructive coronary arteries (INOCA), in which CFR ≤ 2 and IMR > 25 are consensus-recommended thresholds that have not been specifically validated in acute TTS. Based on the combination of both parameters, two CMD patterns were further distinguished in each territory: Pattern A, defined as CFR ≤ 2 with IMR ≤ 25, and Pattern B, defined as CFR ≤ 2 with IMR > 25; these correspond, respectively, to the “functional” and “structural” CMD subtypes originally defined in the INOCA framework [12,16]. Because the INOCA classification was developed for chronic, stable disease, these labels are used in the present study strictly as descriptive terms for the observed combinations of CFR and IMR; their biological equivalence in acute, reversible TTS has not been validated, and Pattern B (the “structural” subtype) must not be interpreted as evidence of permanent microvascular remodelling in this setting. Data quality was ensured by repeating measurements in cases of suboptimal thermodilution curves and by excluding measurements with obvious technical errors.

### 2.3. Statistical Analysis

Continuous variables were summarised using the number of observations, mean, standard deviation, median, and 25th and 75th percentiles. Categorical variables were expressed as absolute and relative frequencies. Analyses were performed for the overall cohort and were further stratified according to the study groups, particularly the AP and MV phenotypes.

Derived variables characterising the presence and type of CMD in individual coronary territories were created based on CFR and IMR values. In addition, the territorial distribution of CMD between the LAD and LCx/OM/RI territories was assessed. Selected indicators were further converted into binary variables and compared between the observed phenotypes.

The statistical significance of between-group differences was assessed according to the type of variable and the nature of the data being compared. For categorical variables, the chi-square test or Fisher’s exact test was used, particularly for contingency tables with low expected frequencies. For continuous variables, the non-parametric Mann–Whitney U test was used; correlations were assessed with the Spearman rank coefficient, and comparisons of a continuous variable across more than two groups with the Kruskal–Wallis test. The level of statistical significance was set at α = 0.05. A total of 54 between-group comparisons were performed across Table 1, Table 2, Table 3, Table 4 and Table 5. Given the exploratory, hypothesis-generating design and the small sample size, no formal correction for multiple testing (e.g., Bonferroni or Benjamini–Hochberg) was applied, as such correction would be overly conservative for a pilot study not intended for confirmatory inference; accordingly, all *p*-values—particularly borderline values close to 0.05—should be interpreted as hypothesis-generating and treated with caution. Generative artificial intelligence (Claude; Anthropic, PBC, San Francisco, CA, USA) was used during the preparation of this manuscript to assist with English-language editing and with the preparation and checking of the figures and tables. It was not used for study design, data collection, or data analysis. All AI-assisted output was reviewed and verified by the authors, who take full responsibility for the content of the manuscript.

Data processing and statistical analyses were performed using the R programming language in the RStudio environment, R version 4.4.2 (31 October 2024 ucrt), with the ggplot2, tidyr, lubridate, dplyr, and gtsummary packages.

## 3. Results

### 3.1. Baseline Characteristics

Between March 2018 and October 2022, 31 patients with TTS (29 women and 2 men), with a median age of 71.0 years (IQR, 65.5–75.5), were enrolled and underwent invasive coronary microcirculatory testing during the acute phase of the disease. The apical phenotype of TTS was present in 20 patients (64.5%), whereas the midventricular phenotype was diagnosed in 11 patients (35.5%). The basal and focal TTS phenotypes were not represented in our cohort. There were no significant differences between the two groups (AP vs. MV) in cardiovascular risk factors or comorbidities; however, the groups differed significantly in age, with patients with the MV phenotype being older than those with the AP phenotype (median, 75 vs. 67 years; *p* = 0.02). The median time from symptom onset to coronary angiography was 6.0 h (IQR, 3.5–27.8 h; range, 1–144 h) for the entire cohort. Overall, 22 patients (71.0%) underwent catheterisation within 24 h of symptom onset, whereas in 9 patients (29.0%), the interval was longer than 24 h. No statistically significant difference in this interval was observed between the two phenotypes. The baseline characteristics of the cohort are shown in Table 1.

**Table 1:** Baseline characteristics by TTS phenotype.

Variable	Entire Cohort (*n* = 31)	Apical TTS (*n* = 20)	Midventricular TTS (*n* = 11)	*p*-Value
**Demographics**				
Age, years, median [IQR]	71.0 [65.5–75.5]	67.0 [61.0–74.0]	75.0 [72.5–77.5]	0.02
Female sex, *n* (%)	29 (93.5)	19 (95.0)	10 (90.9)	1.00
Male sex, *n* (%)	2 (6.5)	1 (5.0)	1 (9.1)	1.00
Time from symptom onset to CAG, h, median [IQR]	6.0 [3.5–27.8]	5.3 [3.0–32.1]	8.5 [4.5–21.0]	0.63
**Cardiovascular risk factors, *n* (%)**				
Hypertension	20 (64.5)	12 (60.0)	8 (72.7)	0.75
Dyslipidaemia	18 (58.1)	13 (65.0)	5 (45.5)	0.50
Type 2 diabetes mellitus	7 (22.6)	5 (25.0)	2 (18.2)	1.00
Smoking	7 (22.6)	5 (25.0)	2 (18.2)	1.00
**Comorbidities, *n* (%)**				
Non-obstructive coronary atherosclerosis (plaque < 30%) ^1^	20 (64.5)	14 (70.0)	6 (54.5)	0.42
COPD/bronchial asthma	6 (19.4)	3 (15.0)	3 (27.3)	0.72
Depression/anxiety	6 (19.4)	3 (15.0)	3 (27.3)	0.72
Systemic disease/CNS comorbidity	7 (22.6)	5 (25.0)	2 (18.2)	1.00

Data are expressed as median [IQR] or number (%). *p*-values were calculated using the Mann–Whitney U test for continuous variables and the chi-square test or Fisher’s exact test for categorical variables. ^1^ Non-obstructive plaques were defined as stenosis < 30%, excluding obstructive coronary artery disease according to the InterTAK criteria. IQR, interquartile range; CAG, coronary angiography; COPD, chronic obstructive pulmonary disease; CNS, central nervous system; TTS, Takotsubo syndrome.

### 3.2. ECG, Laboratory, and Echocardiographic Findings

Baseline ECG was abnormal in all enrolled patients. The most common findings were ST-segment elevation in 14 patients (45.2%) and negative T waves in 15 patients (48.4%); a prolonged QTc interval was recorded at baseline in 4 patients (12.9%). No statistically significant differences were found between the apical and midventricular phenotypes in any of the assessed ECG parameters. Malignant ventricular arrhythmia was recorded in 1 patient with the midventricular phenotype (1/11; 9.1%). The median high-sensitivity cardiac troponin T (hs-cTnT) level was 321 ng/L (IQR, 122–510), with no significant difference between phenotypes (382 vs. 260 ng/L; *p* = 0.636). N-terminal pro-B-type natriuretic peptide (NT-proBNP) levels were 2923 vs. 3153 pg/mL (*p* = 0.669), and C-reactive protein (CRP; *n* = 28) levels were 3.1 vs. 5.8 mg/L (*p* = 0.442); the groups were comparable for both parameters.

Echocardiography performed during the acute phase demonstrated reduced left ventricular systolic function, with a median left ventricular ejection fraction (LVEF) of 45% (IQR, 39.5–50) in the entire cohort. Patients with the apical phenotype had numerically lower LVEF than those with the midventricular phenotype (42% [37–46] vs. 50% [43–52]); however, this difference was not statistically significant (*p* = 0.146). Detailed clinical and echocardiographic parameters are summarised in Table 2.

**Table 2:** Clinical and laboratory characteristics at admission, stratified by TTS phenotype.

Variable	Entire Cohort (*n* = 31)	Apical TTS (*n* = 20)	Midventricular TTS (*n* = 11)	*p*-Value
**ECG findings at admission, *n* (%)**				
Pathological ECG	31 (100.0)	20 (100.0)	11 (100.0)	—
ST-segment elevation	14 (45.2)	9 (45.0)	5 (45.5)	0.999
ST-segment depression	3 (9.7)	1 (5.0)	2 (18.2)	0.580
Negative T waves	15 (48.4)	9 (45.0)	6 (54.5)	0.894
Prolonged QTc interval	4 (12.9)	3 (15.0)	1 (9.1)	0.999
AV block	1 (3.2)	1 (5.0)	0 (0.0)	0.999
Bundle branch block	1 (3.2)	1 (5.0)	0 (0.0)	0.999
Malignant ventricular arrhythmia	1 (3.2)	0 (0.0)	1 (9.1)	0.758
**Biomarkers, median [IQR]**				
hs-cTnT (ng/L) ^1^	321 [122–510]	382 [125–471]	260 [163–541]	0.636
NT-proBNP (pg/mL) ^2^	3084 [1609–4724]	2923 [2492–4698]	3153 [765–5205]	0.669
CRP (mg/L) ^3^	3.9 [1.0–15.7]	3.1 [1.5–12.8]	5.8 [2.3–26.2]	0.442
**Echocardiography at admission, median [IQR]**				
LVEF (%)	45 [39.5–50]	42 [37–46]	50 [43–52]	0.146

Data are expressed as median [IQR] or number (%). *p*-values were calculated using the Mann–Whitney U test for continuous variables and the chi-square test or Fisher’s exact test for categorical variables. ^1^ hs-cTnT was available in 30/31 patients (19 AP, 11 MV). ^2^ NT-proBNP was available in 29/31 patients (18 AP, 11 MV). ^3^ CRP was available in 28/31 patients (18 AP, 10 MV). LVEF, left ventricular ejection fraction; hs-cTnT, high-sensitivity cardiac troponin T; NT-proBNP, N-terminal pro-B-type natriuretic peptide; CRP, C-reactive protein; IQR, interquartile range; AV, atrioventricular; QTc, corrected QT interval.

### 3.3. Invasive Assessment of Coronary Physiology and Microcirculation

Complete invasive assessment was performed in all 31 patients. When present, coronary atherosclerosis in the interrogated vessels was limited to non-obstructive plaques (<30% diameter stenosis). Such plaques were present in 20/31 patients (64.5%), with no difference between the AP and MV groups (*p* = 0.420). No patient had a stenosis ≥ 30%. Coronary dominance was right in 22 patients (71.0%), left in 6 (19.4%), and co-dominant in 3 (9.7%); the proportion of right- versus non-right-dominant patients did not differ significantly between phenotypes (Fisher’s exact test, *p* = 0.217). FFR values were within the normal range in all cases: the median FFR was 0.90 in the LAD territory (IQR, 0.88–0.94; *n* = 31) and 0.99 in the LCx/OM/RI territory (IQR, 0.97–1.00; *n* = 31). Haemodynamically significant epicardial disease was thus excluded as a cause of acute LV dysfunction.

Invasive assessment of microcirculation showed marked heterogeneity and a high prevalence of pathological findings. The median CFR was 1.8 in the LAD territory (IQR, 1.4–3.0) and 1.5 in the LCx/OM/RI territory (IQR, 1.1–2.7). Reduced CFR in the LAD territory, defined as CFR ≤ 2, was observed in 19/31 patients (61.3%), and reduced CFR in the LCx/OM/RI territory was observed in 17/31 patients (54.8%); 24 of 31 patients (77.4%) had a pathological CFR value in at least one territory. IMR values also showed a wide range, with the pathological threshold frequently exceeded: the median IMR was 26.5 in the LAD territory (IQR, 13.6–47.1; *n* = 31) and 19.6 in the LCx/OM/RI territory (IQR, 14.4–37.6; *n* = 31). A pathologically elevated IMR (>25) was present in 16/31 patients (51.6%) in the LAD territory and in 12/31 patients (38.7%) in the LCx/OM/RI territory; 18/31 patients (58.1%) had a pathological IMR value in at least one territory. In summary, invasive measurements performed in the absence of haemodynamically significant epicardial lesions revealed a high prevalence of CMD: at least one pathological parameter, defined as CFR ≤ 2 and/or IMR > 25, was present in 26 of 31 patients (83.9%) (Table 3).

**Table 3:** Invasive assessment of coronary physiology, stratified by TTS phenotype.

Variable	Entire Cohort (*n* = 31)	Apical TTS (*n* = 20)	Midventricular TTS (*n* = 11)	*p*-Value
**Fractional flow reserve (FFR), median [IQR]**				
FFR in the LAD territory	0.90 [0.88–0.94]	0.89 [0.87–0.94]	0.92 [0.89–0.93]	0.868
FFR in the LCx/OM/RI territory	0.99 [0.97–1.00]	0.98 [0.97–1.00]	1.00 [0.97–1.00]	0.245
**Coronary flow reserve (CFR)**				
CFR in the LAD territory, median [IQR]	1.8 [1.4–3.0]	1.6 [1.2–1.8]	2.6 [2.5–3.8]	0.003
CFR ≤2 in the LAD territory, *n* (%)	19 (61.3)	17 (85.0)	2 (18.2)	0.001
CFR in the LCx/OM/RI territory, median [IQR]	1.5 [1.1–2.7]	1.6 [1.1–2.5]	1.5 [1.1–3.1]	0.679
CFR ≤2 in the LCx/OM/RI territory, *n* (%)	17 (54.8)	11 (55.0)	6 (54.5)	1.000
**Index of microcirculatory resistance (IMR)**				
IMR in the LAD territory, median [IQR]	26.5 [13.6–47.1]	28.7 [13.3–48.8]	22.3 [13.8–37.7]	0.680
IMR >25 in the LAD territory, *n* (%)	16 (51.6)	11 (55.0)	5 (45.5)	0.716
IMR in the LCx/OM/RI territory, median [IQR]	19.6 [14.4–37.6]	19.6 [15.2–35.9]	16.8 [13.5–34.4]	0.591
IMR >25 in the LCx/OM/RI territory, *n* (%)	12 (38.7)	8 (40.0)	4 (36.4)	1.000
**Summary indicators of coronary microvascular dysfunction (CMD)**				
CFR ≤2 in at least one territory, *n* (%)	24 (77.4)	17 (85.0)	7 (63.6)	0.210
IMR >25 in at least one territory, *n* (%)	18 (58.1)	12 (60.0)	6 (54.5)	1.000
At least 1 pathological parameter ^1^, *n* (%)	26 (83.9)	18 (90.0)	8 (72.7)	0.317

Data are expressed as median [IQR] or number (%). *p*-values were calculated using the Mann–Whitney U test for continuous variables and the chi-square test or Fisher’s exact test for categorical variables. FFR was ≥0.80 in both territories in all patients, excluding haemodynamically significant epicardial disease. ^1^ CFR ≤ 2 and/or IMR > 25 in any territory. LAD, left anterior descending artery; LCx/OM/RI, left circumflex artery/obtuse marginal branch/ramus intermedius; CFR, coronary flow reserve; IMR, index of microcirculatory resistance; FFR, fractional flow reserve; IQR, interquartile range.

### 3.4. Comparison of Apical and Midventricular Forms of TTS

**Figure 1:**
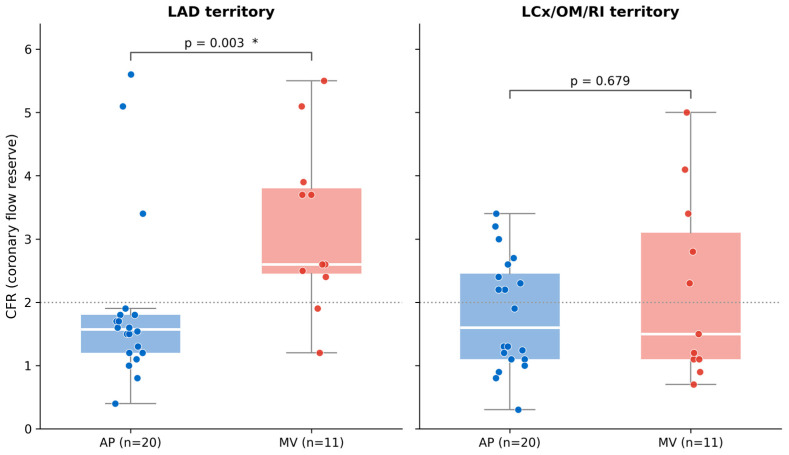
Comparison of CFR values in the LAD territory (**left**) and the LCx/OM/RI territory (**right**) between the apical (AP, blue) and midventricular (MV, red) TTS phenotypes. The boxplot shows the median, interquartile range (IQR), and whiskers (1.5 × IQR); dots represent individual patient values. The dotted line indicates the CFR threshold of 2. * *p* < 0.05. AP, apical phenotype; MV, midventricular phenotype; CFR, coronary flow reserve; LAD, left anterior descending artery; LCx/OM/RI, left circumflex artery/obtuse marginal branch/ramus intermedius; IQR, interquartile range; TTS, Takotsubo syndrome.

When patients were compared according to phenotype (AP vs. MV), significant differences were found in microcirculatory parameters, particularly in the LAD territory. In the apical phenotype, the median CFR in the LAD territory was 1.6 (IQR, 1.2–1.8), and CFR ≤ 2 was recorded in 17/20 patients (85.0%). In the midventricular phenotype, CFR values in the LAD territory were significantly higher (median, 2.6; IQR, 2.5–3.8), and CFR ≤ 2 was recorded in only 2/11 patients (18.2%). The difference in CFR values between phenotypes was statistically significant (*p* = 0.003; Figure 1). Conversely, in the LCx/OM/RI territory, no significant differences in CFR values were found between phenotypes (*p* = 0.679): the median CFR was 1.6 (IQR, 1.1–2.5) in the apical phenotype and 1.5 (IQR, 1.1–3.1) in the midventricular phenotype, and the proportion of patients with reduced CFR in this territory was virtually identical (11/20, 55.0% vs. 6/11, 54.5%; *p* = 1.000) (Table 3).

**Figure 2:**
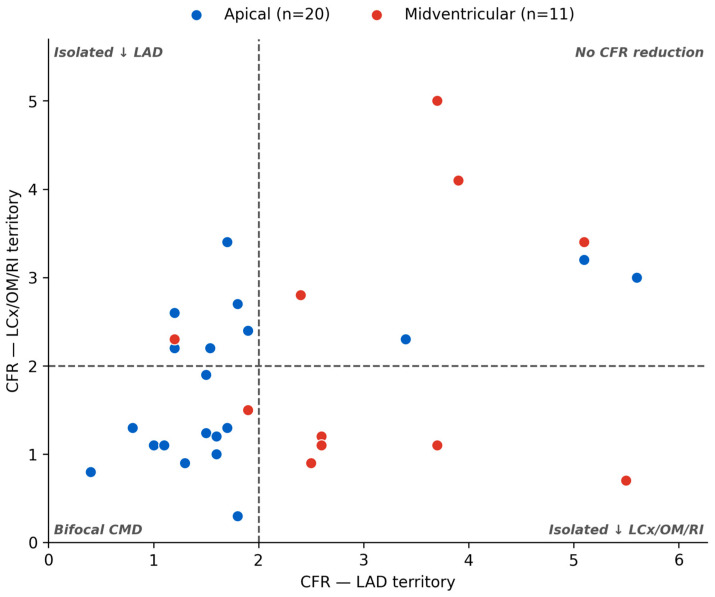
Relationship between CFR values in the LAD territory (*x*-axis) and the LCx/OM/RI territory (*y*-axis) in individual patients. Dashed lines indicate the CFR threshold of 2 and delineate four quadrants corresponding to the patterns of territorial distribution of reduced CFR. The downward arrow (↓) denotes reduced CFR (≤2). AP, apical phenotype; MV, midventricular phenotype; CFR, coronary flow reserve; LAD, left anterior descending artery; LCx/OM/RI, left circumflex artery/obtuse marginal branch/ramus intermedius; TTS, Takotsubo syndrome.

Although the overall prevalence of reduced CFR in the LCx/OM/RI territory was comparable across phenotypes, analysis of the territorial distribution of involvement revealed a different pattern. In the apical phenotype, reduced CFR was present simultaneously in both the LAD and LCx/OM/RI territories in 11/20 patients (55.0%), whereas isolated CFR reduction in the LCx/OM/RI territory without LAD involvement was not observed in any patient (0/20; 0.0%). In contrast, in the midventricular phenotype, isolated LCx/OM/RI involvement without concurrent CFR reduction in the LAD territory predominated, being observed in 5/11 patients (45.5%), whereas simultaneous involvement of both territories was present in only 1/11 patients (9.1%). These differences were statistically significant for isolated LCx/OM/RI involvement (45.5% vs. 0.0%; *p* = 0.003) and simultaneous involvement of both territories (55.0% vs. 9.1%; *p* = 0.020) (Table 4, Figure 2).

**Table 4:** Subtype classification of CMD and territorial distribution of involvement in the LAD and LCx/OM/RI territories.

Variable	Entire Cohort (*n* = 31)	Apical TTS (*n* = 20)	Midventricular TTS (*n* = 11)	*p*-Value
**A. Subtype classification of CMD in the LAD territory**				
Pattern B (CFR ≤ 2, IMR > 25)	11 (35.5)	10 (50.0)	1 (9.1)	0.047
Pattern A (CFR ≤ 2, IMR ≤ 25)	8 (25.8)	7 (35.0)	1 (9.1)	0.203
Isolated IMR elevation (CFR > 2 and IMR > 25)	5 (16.1)	1 (5.0)	4 (36.4)	0.042
No CMD in LAD (CFR > 2 and IMR ≤ 25)	7 (22.6)	2 (10.0)	5 (45.5)	0.067
**B. Subtype classification of CMD in the LCx/OM/RI territory**				
Pattern B (CFR ≤ 2, IMR > 25)	9 (29.0)	6 (30.0)	3 (27.3)	1.000
Pattern A (CFR ≤ 2, IMR ≤ 25)	8 (25.8)	5 (25.0)	3 (27.3)	1.000
Isolated IMR elevation (CFR > 2 and IMR > 25)	3 (9.7)	2 (10.0)	1 (9.1)	1.000
No CMD in LCx/OM/RI (CFR > 2 and IMR ≤ 25)	11 (35.5)	7 (35.0)	4 (36.4)	1.000
**C. Territorial distribution of CMD (definition: CFR ≤ 2)**				
CMD in both territories (LAD and LCx/OM/RI)	12 (38.7)	11 (55.0)	1 (9.1)	0.020
CMD in LAD territory only	7 (22.6)	6 (30.0)	1 (9.1)	0.372
CMD in LCx/OM/RI territory only	5 (16.1)	0 (0.0)	5 (45.5)	0.003
No CMD in either territory	7 (22.6)	3 (15.0)	4 (36.4)	0.210

Data are expressed as number (%). *p*-values were calculated using Fisher’s exact test for comparison between AP and MV phenotypes. Sections A and B show the subtype classification of CMD based on the combination of CFR and IMR in each territory (*n* = 31). Section C shows the territorial distribution of CMD, defined exclusively on the basis of CFR. CMD, coronary microvascular dysfunction; CFR, coronary flow reserve; IMR, index of microcirculatory resistance; LAD, left anterior descending artery; LCx/OM/RI, left circumflex artery/obtuse marginal branch/ramus intermedius. Pattern A and Pattern B correspond, respectively, to the functional and structural CMD subtypes of the INOCA framework and are used here for descriptive purposes only.

IMR values showed a wide range, with a trend towards higher values in the apical phenotype; however, neither median IMR values nor the proportion of pathologically elevated values differed significantly between phenotypes. In the LAD territory, the median IMR was 28.7 (IQR, 13.3–48.8) in the apical phenotype compared with 22.3 (IQR, 13.8–37.7) in the midventricular phenotype (*p* = 0.680). In the LCx/OM/RI territory, the median IMR was 19.6 (IQR, 15.2–35.9) in the apical phenotype and 16.8 (IQR, 13.5–34.4) in the midventricular phenotype (*p* = 0.591). The proportion of patients with pathologically elevated IMR also did not differ significantly between phenotypes (LAD: 11/20, 55.0% vs. 5/11, 45.5%; *p* = 0.716; LCx/OM/RI: 8/20, 40.0% vs. 4/11, 36.4%; *p* = 1.000). A notable phenotype-specific feature was isolated IMR elevation > 25 with preserved CFR in the LAD territory, which was significantly more common in the midventricular phenotype than in the apical phenotype (4/11, 36.4% vs. 1/20, 5.0%; *p* = 0.042) (Table 4).

**Figure 3:**
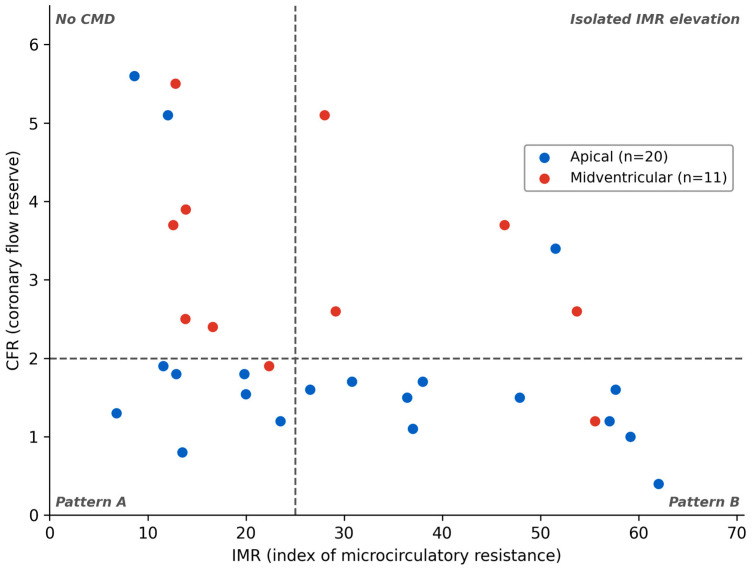
Distribution of individual IMR (*x*-axis) and CFR (*y*-axis) values in the LAD territory according to TTS phenotype. Dashed lines indicate the thresholds of CFR = 2 and IMR = 25, delineating four CMD subtypes. No CMD: CFR > 2 and IMR ≤ 25; isolated IMR elevation: CFR > 2 and IMR > 25; Pattern A: CFR ≤ 2 and IMR ≤ 25; Pattern B: CFR ≤ 2 and IMR > 25. AP, apical phenotype; MV, midventricular phenotype; CFR, coronary flow reserve; IMR, index of microcirculatory resistance; CMD, coronary microvascular dysfunction.

**Figure 4:**
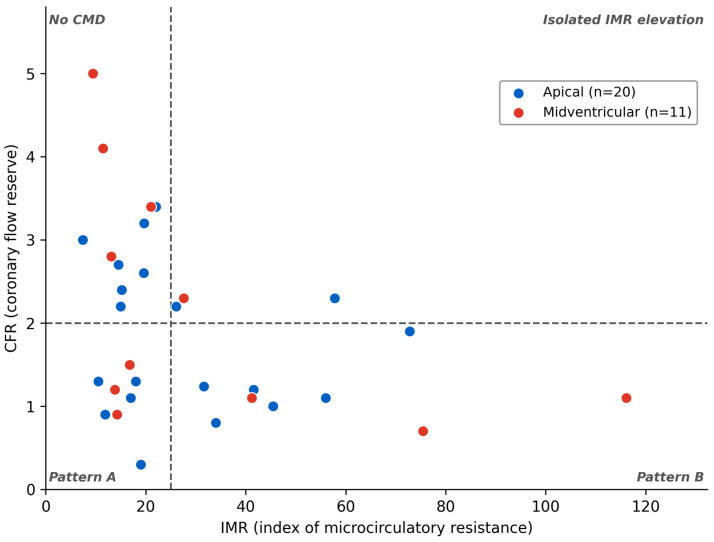
Distribution of individual IMR (*x*-axis) and CFR (*y*-axis) values in the LCx/OM/RI territory according to TTS phenotype. Dashed lines indicate the thresholds of CFR = 2 and IMR = 25, delineating four CMD subtypes. No CMD: CFR > 2 and IMR ≤ 25; isolated IMR elevation: CFR > 2 and IMR > 25; Pattern A: CFR ≤ 2 and IMR ≤ 25; Pattern B: CFR ≤ 2 and IMR > 25. AP, apical phenotype; MV, midventricular phenotype; CFR, coronary flow reserve; IMR, index of microcirculatory resistance; CMD, coronary microvascular dysfunction.

**Figure 5:**
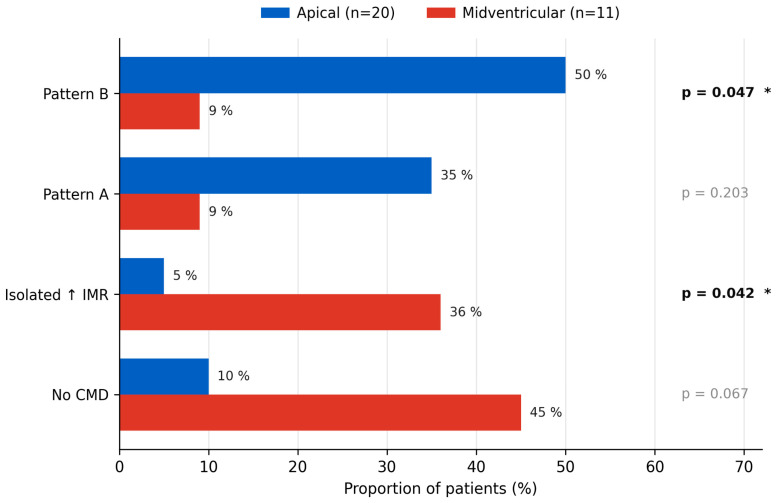
Distribution of CMD subtypes in the LAD territory according to TTS phenotype. Values are expressed as the proportion of patients (%) in each group. The asterisk (*) indicates a statistically significant difference between phenotypes (*p* < 0.05); all *p*-values are unadjusted and should be interpreted as exploratory. Subtype analysis of the LCx/OM/RI territory showed no phenotype-specific differences in any of the four categories (all *p* = 1.000). The upward arrow (↑) denotes elevated IMR (>25). AP, apical phenotype; MV, midventricular phenotype; CMD, coronary microvascular dysfunction; IMR, index of microcirculatory resistance.

In the LAD territory, the apical phenotype had a significantly higher proportion of Pattern B than the midventricular phenotype (10/20, 50.0% vs. 1/11, 9.1%; *p* = 0.047). The proportions of Pattern A (7/20, 35.0% vs. 1/11, 9.1%; *p* = 0.203) and the no-CMD category (2/20, 10.0% vs. 5/11, 45.5%; *p* = 0.067) did not differ significantly between phenotypes. In the LCx/OM/RI territory, no phenotype-related differences were found in any of the four subtype categories (all *p* = 1.000). Complete subtype analysis of both territories is presented in Table 4; the individual CFR–IMR distributions delineating the four CMD patterns are shown for the LAD and LCx/OM/RI territories in Figure 3 and Figure 4, respectively, and the LAD subtype proportions are illustrated in Figure 5.

To assess whether these phenotype differences were confounded by the timing of assessment, we examined the symptom-to-catheterisation interval against CFR, IMR, and the CMD subtype classification: no significant association was found in either territory, whether the interval was analysed continuously (Spearman ρ −0.02 to +0.10) or dichotomised at 24 or 6 h (all *p* ≥ 0.28), and the subtype distribution was independent of timing (*p* = 0.93). In a sensitivity analysis restricted to the 22 patients assessed within 24 h, the principal phenotype differences persisted (LAD-CFR remained lower in the apical phenotype, median 1.6 vs. 2.55; isolated LCx/OM/RI involvement remained more frequent in the midventricular phenotype, 4/8 vs. 0/14, *p* = 0.010). These analyses provided no evidence that the observed phenotype-related differences were driven by the timing of invasive assessment.

### 3.5. Twelve-Week Follow-Up

The 12-week follow-up clinical examination was completed in 25 of 31 patients (80.6%) during an outpatient visit at our centre, during which follow-up echocardiography was also performed. For two additional patients who did not attend the scheduled clinical visit, follow-up echocardiographic data obtained at external cardiology centres were used for analysis. Follow-up echocardiography was therefore available for a total of 27 of 31 patients (87.1%). Of the four patients without follow-up echocardiography, one died from a non-cardiac cause before the scheduled visit, and three did not attend for unknown reasons and were classified as lost to follow-up.

**Figure 6:**
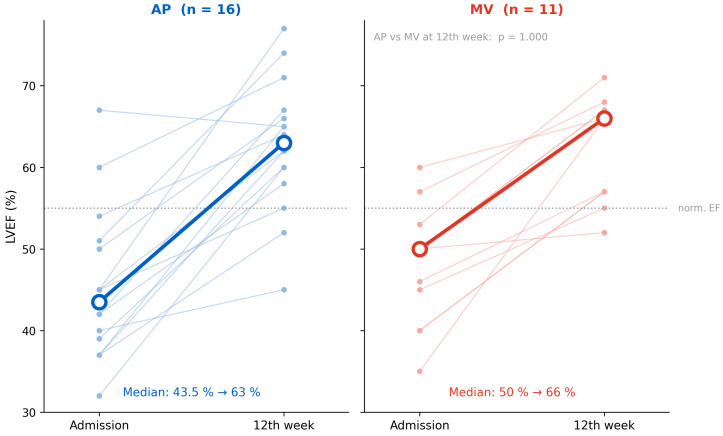
Individual trajectories of left ventricular ejection fraction (LVEF) from admission to week 12 in patients with apical (AP, **left**, blue) and midventricular (MV, **right**, red) TTS phenotypes. The bold line indicates the group median; open symbols indicate median values at each time point. The dotted line indicates the lower limit of normal LVEF (55%). Admission LVEF is shown only for patients with complete follow-up data (AP: *n* = 16; MV: *n* = 11); therefore, the AP admission median (43.5%) differs slightly from that shown in Table 2 (42%), which includes all 20 patients with the AP phenotype. AP, apical phenotype; MV, midventricular phenotype; LVEF, left ventricular ejection fraction; TTS, Takotsubo syndrome.

Follow-up echocardiography confirmed normalisation of median LV systolic function in both phenotypes, with a median LVEF of 63% (60–66) in patients with apical TTS (*n* = 16) and 66% (57–67) in patients with midventricular TTS (*n* = 11; *p* = 1.000; Figure 6). Overall mortality during the follow-up period was therefore 3.2% (1/31; AP, 1/20 [5.0%] vs. MV, 0/11 [0.0%]; *p* = 1.000). No patient was readmitted for a cardiovascular event or required repeat coronary angiography. Persistent dyspnoea was reported in 9 of 25 evaluable patients (36.0%), with no significant difference between the apical and midventricular phenotypes (5/15 [33.3%] vs. 4/10 [40.0%]; *p* = 1.000). Intermittent angina symptoms were recorded in 1 patient with the midventricular phenotype (AP, 0/15 [0.0%] vs. MV, 1/10 [10.0%]; *p* = 0.400) (Table 5).

**Table 5:** 12-week clinical and echocardiographic follow-up.

Variable	Entire Cohort (*n* = 31)	Apical TTS (*n* = 20)	Midventricular TTS (*n* = 11)	*p*-Value
**Follow-up availability, *n* (%)**				
Clinical examination at week 12 completed	25 (80.6)	15 (75.0)	10 (90.9)	0.388
Follow-up echocardiography available	27 (87.1)	16 (80.0)	11 (100.0)	0.267
**Left ventricular function at week 12, median [IQR]**				
LVEF (TTE) (%) ^1^	64 [57–67]	63 [60–66]	66 [57–67]	1.000
**Clinical events during 12-week follow-up, *n* (%)**				
Mortality from non-cardiac cause	1 (3.2)	1 (5.0)	0 (0.0)	1.000
Cardiovascular rehospitalisation	0 (0.0)	0 (0.0)	0 (0.0)	—
Repeat coronary angiography	0 (0.0)	0 (0.0)	0 (0.0)	—
**Symptoms at week 12 in evaluable patients ^2^, *n* (%)**				
Angina pectoris	1/25 (4.0)	0/15 (0.0)	1/10 (10.0)	0.400
Dyspnoea	9/25 (36.0)	5/15 (33.3)	4/10 (40.0)	1.000

Data are expressed as median [IQR] or number (%). *p*-values were calculated using the Mann–Whitney U test for continuous variables and the chi-square test or Fisher’s exact test for categorical variables. ^1^ LVEF at week 12 was available in 27/31 patients (16 AP, 11 MV). ^2^ Clinical follow-up was available in 25/31 patients (15 AP, 10 MV); the denominator indicates the number of evaluable patients in each group. TTE, transthoracic echocardiography; LVEF, left ventricular ejection fraction; IQR, interquartile range.

## 4. Discussion

In this prospective single-centre study, we assessed epicardial and microvascular coronary function in patients with TTS during the acute phase of the disease using invasive coronary physiology. Epicardial flow was preserved in all examined coronary territories, confirming that acute left ventricular systolic dysfunction in TTS was not caused by haemodynamically significant epicardial coronary stenosis. Conversely, microcirculatory parameters showed a high prevalence of pathological values and marked heterogeneity. At least one abnormal microvascular parameter was present in 26 of 31 patients (83.9%), supporting the hypothesis that CMD plays a significant role in the pathophysiology of TTS [17,18,19].

When TTS phenotypes were compared, a significant difference was observed particularly in the LAD territory, where the apical phenotype was characterised by markedly lower CFR than the midventricular phenotype. In the LCx/OM/RI territory, no significant differences were found between phenotypes in absolute CFR or IMR values; however, analysis of the territorial distribution of involvement revealed a distinct pattern. IMR measurements in the apical phenotype showed a non-significant trend towards higher values in both territories; the exception was isolated IMR elevation with preserved CFR in the LAD territory, which was significantly more common in the midventricular phenotype. Another important observation was the marked heterogeneity of CFR and IMR combinations in the LAD territory in the apical phenotype.

It should be emphasised that the reported prevalence of CMD in TTS varies substantially across published studies, depending on the diagnostic method used, the definition of abnormality, and the cut-off values selected for individual indices. These differences make direct comparison of absolute numerical data between cohorts difficult [20]. The high prevalence of CMD observed in our cohort is consistent with the results of recent studies evaluating invasive coronary physiology in patients with TTS. For example, Shetrit et al. demonstrated the presence of at least one pathological microvascular parameter in approximately 83% of patients with TTS, which is almost identical to the prevalence observed in our cohort. The authors also showed that the time interval between symptom onset and invasive testing can significantly influence CFR values, with longer intervals being associated with higher CFR values. In our cohort, the median time from symptom onset to catheterisation was 6.0 h (IQR, 3.5–27.8 h), with considerable variability (range, 1–144 h); therefore, the inclusion of patients with longer intervals may have contributed to greater heterogeneity in CFR values across the entire cohort and should be taken into account when interpreting the results. It should be noted that Shetrit et al. used a less stringent CFR threshold (<2.5 vs. ≤2.0 in our study); nevertheless, the prevalence of CMD in both cohorts was virtually identical, further supporting the significance of this finding [18]. Rivero et al. similarly reported an inverse relationship between IMR and the interval from symptom onset to invasive measurement in a small TTS cohort, supporting a time-dependent recovery of microvascular resistance during the acute phase [21]. To directly address this potential confounder in our cohort, we tested the association between the symptom-to-catheterisation interval and each microvascular parameter and found no significant relationship (Section 3.4), with no evidence that the observed phenotype-related differences were driven by the timing of assessment. Moreover, because a time-dependent recovery would be expected to lower IMR and raise CFR in later-assessed patients, the presence of Pattern B (elevated IMR with reduced CFR) even in patients catheterised late argues against a timing-driven artefact.

Similarly, Ekenbäck et al. demonstrated the presence of microvascular dysfunction in approximately 78% of patients with TTS and reported lower CFR in the apical phenotype than in other forms of the syndrome, whereas differences in IMR were not statistically significant [19]. Our results support and further extend these findings by analysing differences between individual coronary territories. Other studies suggest that microvascular resistance may have prognostic significance. Sans-Roselló et al. demonstrated that the extent of increased microvascular resistance, measured using the NH-IMRangio index (non-hyperaemic, angiographically derived microvascular resistance index), was associated with a higher risk of major adverse cardiovascular events (MACE) during 1-year follow-up [22]. Similar conclusions were reached in the pooled individual-patient-data analysis by Eerdekens et al., in which elevated IMR was an independent predictor of both mortality and major adverse cardiovascular and cerebrovascular events [23]. These findings support the importance of detailed microcirculatory assessment in TTS and, although our study was not powered for clinical outcomes, raise the hypothesis that the phenotype-specific patterns observed here—particularly the higher prevalence of elevated IMR (Pattern B) in the LAD territory in apical TTS—could carry prognostic relevance, warranting prospective evaluation. Imaging studies using positron emission tomography (PET) further demonstrate that microvascular dysfunction may persist even after normalisation of left ventricular systolic function. For example, Kadoya et al. described global CMD with persistent regional perfusion abnormalities even after clinical recovery [24].

The results of our study suggest that microvascular dysfunction in TTS may not be diffuse but rather exhibits a phenotype-dependent territorial distribution. The apical phenotype was characterised by a predominance of microcirculatory impairment in the LAD territory, often with concomitant involvement of the LCx/OM/RI territory, representing a bifocal pattern with LAD dominance. In contrast, in the midventricular phenotype, isolated impairment of the LCx/OM/RI territory predominated, with relatively preserved microcirculatory function in the LAD territory. This distinct topographical pattern is consistent with territorially heterogeneous microcirculatory impairment, possibly related to the regional distribution of adrenergic receptors and differing myocardial sensitivity to catecholamine stress [25,26,27,28]. This territorial pattern is unlikely to be an artefact of coronary dominance: the LCx/OM/RI territory represents the lateral/anterolateral wall in every dominance pattern, our cohort was predominantly right-dominant (Section 3.3), and the isolated LCx/OM/RI involvement in the midventricular phenotype persisted when the analysis was restricted to right-dominant patients (isolated LCx/OM/RI involvement 4/6 vs. 0/16, *p* = 0.002; LAD-CFR 1.65 vs. 2.55, *p* = 0.022).

In addition to territorial distribution, our study provides insight into the qualitative nature of microvascular dysfunction, namely the patterns of CFR and IMR combinations. Subtype analysis of CMD based on the combination of CFR and IMR was performed in both assessed territories. In the apical phenotype, two dominant combinations were identified in the LAD territory: reduced CFR with normal IMR and reduced CFR with elevated IMR. These correspond to Pattern A and Pattern B (the functional and structural CMD subtypes of the INOCA framework; see Section 2.2), the latter without implying permanent microcirculatory remodelling in this reversible setting [29,30].

In contrast, in the LCx/OM/RI territory all four subtypes were represented without a clear phenotype-specific pattern; the qualitative, subtype-level differences between phenotypes were therefore most evident in the LAD territory.

Clinical data from the 12-week follow-up, available for a subset of the cohort, showed no cardiovascular rehospitalisations or repeat coronary angiography, consistent with the favourable clinical course of haemodynamically stable forms of TTS described in the literature. The recorded non-cardiac mortality rate (3.2%) corresponds to the expected mortality in the elderly population affected by this syndrome. Persistent dyspnoea despite normalisation of median LVEF, reported in roughly one third of patients, is consistent with reports of incomplete microvascular recovery in TTS and raises the possibility that microvascular dysfunction outlasts the recovery of systolic function; however, in the absence of repeat invasive assessment or cardiopulmonary exercise testing, non-cardiac contributors such as deconditioning, age-related comorbidity or pulmonary disease cannot be excluded. This question warrants further investigation.

An important and still unresolved question is whether the microvascular abnormalities observed in TTS are a primary driver of the acute left ventricular dysfunction or, at least in part, a secondary consequence of it. On one hand, microvascular dysfunction has long been proposed as a mechanism underlying the regional wall-motion abnormalities of TTS: the intense catecholamine surge that triggers the syndrome can induce microvascular vasoconstriction and endothelial dysfunction in parallel with direct cardiomyocyte injury, and the prognostic weight of elevated IMR argues against a purely bystander role [8,9,29]. On the other hand, several observations support a secondary or contributory component: microvascular indices tend to normalise in parallel with the recovery of wall motion, and myocardial stunning and interstitial oedema themselves increase resting coronary flow and exert extravascular compression on the microcirculation, both of which can lower CFR and raise IMR independently of intrinsic microvascular disease [31]. Our study cannot disentangle these contributions; nevertheless, the persistence of microvascular dysfunction beyond the recovery of systolic function documented in some patients [24] suggests that CMD is unlikely to be merely a passive consequence of contractile impairment. The most plausible synthesis is that catecholamine excess drives both cardiomyocyte injury and microvascular dysfunction, which subsequently interact and amplify one another, so that CMD in TTS may be best regarded as both cause and consequence rather than either in isolation [31].

### 4.1. Perspectives and Future Directions

The following section interprets our findings in the context of the proposed pathophysiological mechanisms of TTS and considers their potential therapeutic implications; these interpretations are hypothesis-generating rather than confirmatory.

Simultaneous involvement of both territories in apical TTS likely reflects more extensive sympathoadrenal activation extending beyond the apical region; conversely, isolated LCx/OM/RI involvement in midventricular TTS co-localises with the anterolateral midventricular segments that this territory supplies. The apical myocardium has a higher density of β2-adrenergic receptors than the midventricular and basal segments. During massive catecholamine stimulation, β2-receptor signalling at the apex switches to the inhibitory Gi pathway, contributing to a negative inotropic effect at the apex and likely also to impaired vasodilatory capacity of the microcirculation supplied primarily by the LAD territory [25,26].

Pattern A in TTS can be explained by the distinct effects of two vasodilatory pathways. Adenosine, which is used to induce maximal hyperaemia, acts directly on endothelium-independent A2A receptors on vascular smooth muscle cells, thereby bypassing both endothelial and β-adrenergic regulation. This pathway may remain relatively preserved in acute TTS, and IMR measured during hyperaemia therefore does not exceed the pathological threshold [15]. Conversely, reduced CFR is mediated by mechanisms affecting resting coronary flow. In stunned myocardium, resting coronary flow increases because of disruption of normal autoregulation and loss of microvascular vasomotor tone, and the impaired β2-mediated vasodilation described above may further contribute [8,25,26]. Higher resting flow with relatively preserved maximal hyperaemic capacity therefore leads to a reduction in CFR as a ratio, without the microvascular resistance threshold being exceeded. In cases of more severe catecholamine dysregulation, α1-adrenergic vasoconstriction may exceed the level that can be overcome by adenosine-induced hyperaemia, and the pattern may then shift towards Pattern B.

Pattern B, on the other hand, corresponds to microcirculatory injury in the acute to subacute phase of TTS. Its main features include microvascular oedema and interstitial mononuclear inflammatory infiltration, predominantly involving lymphocytes and macrophages [8,9]. Platelet activation with microthrombosis in resistance vessels may also contribute to increased microvascular resistance; however, this has been documented in TTS only in a subset of patients, and its quantitative contribution to IMR elevation remains unclear [9,26]. In TTS, both patterns most likely represent transient, reversible phenomena rather than the irreversible fibrotic remodelling of the microcirculation that follows myocardial infarction; the oedema, inflammation, and microthrombosis underlying Pattern B in particular appear to resolve over time in most patients, consistent with imaging studies documenting gradual resolution of CMD after clinical recovery, although resolution may be incomplete in some patients even after normalisation of left ventricular systolic function [19,24].

The isolated IMR elevation with preserved CFR in the LAD territory that characterised the midventricular phenotype is a recognised discordant pattern in coronary physiology, resulting from a proportional reduction in resting and hyperaemic flow, whereby CFR remains preserved as a ratio despite a true increase in microvascular resistance [12,15]. In midventricular TTS, this pattern could hypothetically be explained by less pronounced stunning of the apical segments or by mechanical compression of resistance vessels due to interstitial oedema extending beyond the midventricular segments into the apical segments supplied by the LAD territory. However, the exact mechanism remains unclear, and its clinical impact in TTS has not yet been validated.

Distinguishing between the two CMD patterns could have practical therapeutic implications. Pattern A, characterised by reduced CFR with preserved hyperaemic microvascular resistance, might hypothetically respond to vasodilator therapy, although this requires prospective evaluation. In contrast, Pattern B, with elevated IMR, likely reflects more severe acute involvement characterised by oedema and inflammation, for which anti-inflammatory strategies warrant investigation.

Prospective validation of these pattern- and territory-specific profiles, ideally incorporating follow-up invasive assessment of the microcirculation and tissue characterisation, will be required before they can guide stratified therapeutic strategies.

### 4.2. Limitations

The thresholds used (CFR ≤ 2, IMR > 25) are based on consensus recommendations for INOCA [12,16] and are consistent with the methodology of comparable studies in TTS [19,23]; however, these thresholds have not yet been specifically validated in the TTS population. By definition, CFR is the ratio of hyperaemic to resting flow, and its values may therefore be influenced during the acute phase of TTS by increased resting flow due to myocardial stunning. This limits the interpretation of absolute CFR differences as a purely microvascular finding.

The territorial distribution of CMD was defined in our study exclusively on the basis of reduced CFR (≤2). Isolated IMR elevation with preserved CFR, which was present in 36.4% of patients with the midventricular phenotype, was not included in this classification; if a combined CMD definition including IMR had been used, this could have modified the interpretation of bifocal versus unifocal patterns of involvement. CMD subtype classification, based on the combination of CFR and IMR, was performed for both assessed territories. The analysis in the LCx/OM/RI territory was exploratory in nature and, given the small number of patients in each category, requires cautious interpretation and validation in future studies with larger sample sizes. A further limitation is the absence of follow-up assessment of microcirculation, which would have allowed characterisation of the dynamics of CMD resolution during convalescence.

The study also did not include acetylcholine provocation testing, which would have enabled assessment of coronary vasospasm and endothelial function and could therefore have contributed to a more accurate classification of the various types of microvascular dysfunction. The study included only haemodynamically stable patients; patients with cardiogenic shock, which complicates the course of TTS in approximately 10% of cases [4], were excluded for practical and safety reasons. The results may therefore not fully reflect the spectrum of microvascular abnormalities in the most severe forms of the disease and may underestimate the overall severity of CMD in the TTS population.

Invasive testing was restricted to the LAD and LCx/OM/RI territories of the left coronary artery. In a small number of initial patients, we attempted three-vessel physiological assessment; however, the additional procedural time required was poorly tolerated, and complete right coronary data were obtained in only a few patients. For patient safety and tolerability, the protocol was therefore limited to the left coronary artery. Consequently, concomitant involvement of the non-interrogated right coronary territory cannot be excluded, and the reported territorial distribution applies specifically to the left coronary circulation. The symptom-to-catheterisation interval was not standardised but showed no significant association with the measured parameters (Section 3.4), making a timing-related bias unlikely.

Cardiac magnetic resonance imaging, which would have linked myocardial oedema to the physiological findings, was not performed; the relative contributions of primary microvascular dysfunction versus stunning- and oedema-related changes therefore remain undetermined and warrant future studies combining invasive physiology with tissue characterisation.

## 5. Conclusions

Invasive assessment of coronary physiology in patients with TTS confirms preserved epicardial coronary function in the setting of a high prevalence of CMD during the acute phase of the disease. Microvascular involvement exhibited a distinct phenotype-specific pattern. Bifocal involvement of both territories, the LAD and LCx/OM/RI territories, characterised the apical phenotype, whereas isolated involvement of the LCx/OM/RI territory (within the interrogated left coronary circulation) predominated in the midventricular phenotype. Within the LAD territory in the apical phenotype, we further distinguished two microvascular patterns, Pattern A (reduced CFR with normal IMR) and Pattern B (reduced CFR with elevated IMR), which may correspond to different forms of acute microvascular involvement. This study therefore provides a phenotype- and topography-specific view of microcirculation in TTS, which could have implications for future stratified therapeutic approaches. These findings should be regarded as hypothesis-generating given the small sample size; nevertheless, they identify consistent phenotype-associated differences in microvascular physiology and provide a rationale for larger, adequately powered studies to confirm their clinical and prognostic relevance.

## Data Availability

The data presented in this study are available on request from the corresponding author. The data are not publicly available due to privacy and ethical restrictions. Jiri Jarkovsky and Andrea Kyselova take responsibility for all aspects of the reliability and freedom from bias of the data presented and their discussed interpretation.

## Abbreviations

The following abbreviations are used in this manuscript:

ACS	Acute Coronary Syndrome
AMI	Acute Myocardial Infarction
AP	Apical Phenotype
CAG	Coronary Angiography
CFR	Coronary Flow Reserve
CMD	Coronary Microvascular Dysfunction
CRP	C-Reactive Protein
ECG	Electrocardiogram
FFR	Fractional Flow Reserve
hs-cTnT	High-Sensitivity Cardiac Troponin T
IMR	Index of Microcirculatory Resistance
INOCA	Ischaemia with Non-Obstructive Coronary Arteries
IQR	Interquartile Range
LAD	Left Anterior Descending Artery
LCx	Left Circumflex Artery
LV	Left Ventricle
LVEF	Left Ventricular Ejection Fraction
MACE	Major Adverse Cardiovascular Events
MV	Midventricular Phenotype
NT-proBNP	N-Terminal Pro-B-Type Natriuretic Peptide
OM	Obtuse Marginal
PET	Positron Emission Tomography
RI	Ramus Intermedius
TTS	Takotsubo Syndrome
